# Convergent and distinctive functions of transcription factors VdYap1, VdAtf1, and VdSkn7 in the regulation of nitrosative stress resistance, microsclerotia formation, and virulence in *Verticillium dahliae*

**DOI:** 10.1111/mpp.12988

**Published:** 2020-09-20

**Authors:** Chen Tang, Xianjiang Jin, Steven J. Klosterman, Yonglin Wang

**Affiliations:** ^1^ Beijing Key Laboratory for Forest Pest Control College of Forestry Beijing Forestry University Beijing China; ^2^ United States Department of Agriculture Agricultural Research Service Salinas California USA

**Keywords:** nitrosative stress, VdAtf1, VdSkn7, VdYap1, *Verticillium dahliae*, virulence

## Abstract

Reactive oxygen/nitrogen species (ROS/RNS) play a fundamental role in plant–fungal interactions. How pathogenic fungi manipulate plant‐derived ROS/RNS is of importance to the outcomes of these interactions. In this study, we explored the individual and combined contributions of three transcription factors, VdAtf1, VdYap1, and VdSkn7, in the response to ROS/RNS, microsclerotia formation, and virulence in the plant wilt pathogen *Verticillium dahliae*. We showed that VdYap1 is essential for ROS response. Additionally, mutants lacking any combination of the three genes shared significant hypersensitivity to nitro‐oxidative stress like sodium nitroprusside dehydrate and double deletions lacking *VdYap1* and *VdAtf1* resulted in further increased sensitivity to ROS. Double deletion of *VdAtf1* and *VdSkn7* reduced melanin production and virulence while simultaneous lack of *VdSkn7* and *VdYap1* disrupted nitrogen metabolism and ROS resistance. Finally, comparison of transcriptional profiles of the respective single or double mutants in response to nitro‐oxidative stress revealed that the three transcription factors are involved in denitrification of nitrated alkanes and lipids to protect against nitro‐oxidative stress. Taken together, our results demonstrate convergent and distinctive functions of VdYap1, VdAtf1, and VdSkn7 in *V. dahliae*, and provide new data on their roles in response to ROS/RNS in fungi.

## Graphical abstract

VdYap1, VdAtf1, and VdSkn7 play convergent and distinctive roles in oxidative and nitrosative resistance, microsclerotia formation, and virulence in *Verticillium dahliae*.

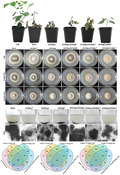

## 1 INTRODUCTION

Vascular wilt disease caused by *Verticillium dahliae* impacts over 200 plant species worldwide and results in losses of agricultural production, as well as damage to ornamental plants and ecological habitats (Klosterman *et al*., 2009). In China, *V. dahliae* has not only posed a major threat to crop production, but has also taken a heavy toll on ornamental perennials like smoke trees (*Cotinus coggygria*), the leaves of which provide beautiful scenery at the Fragrant Hills Park in Beijing, China (Wang *et al*., 2013). *V. dahliae* penetrates plants through the roots via infectious hyphae that germinate from the long‐lived soilborne microsclerotia (Fradin and Thomma, 2006; Klosterman *et al*., 2009). After penetration, the fungus continues to proliferate in the xylem, resulting in characteristic plant wilt symptoms (Beckman *et al*., 1976).

During plant–pathogen interactions, plants rapidly generate reactive oxygen species (ROS) like hydrogen peroxide (H_2_O_2_) and reactive nitrogen species (RNS) like nitric oxide (NO) that are important as signalling molecules and antimicrobial substances to prevent pathogen invasion and spread (Hong *et al*., 2007). For example, *V. dahliae* toxins can generate NO and H_2_O_2_ from cotton suspension cells to induce host responses (Jia *et al*., 2007). These compounds not only mediate plant signalling for several physiological processes, but also give rise to oxidative and nitrosative stress (Besson‐Bard *et al*., 2008; Corpas and Barroso, 2013). H_2_O_2_ production has many adverse biological effects on cellular macromolecules such as lipids, DNA, and proteins, and NO also causes damage through lipid peroxidation and S‐nitrosylation of proteins, which impact cellular activities, ultimately leading to cell death (Apel and Hirt, 2004; Martinez and Andriantsitohaina, 2009; Das and Roychoudhury, 2014). For survival and colonization, pathogens must overcome H_2_O_2_ and NO stresses generated by plants (Segal and Wilson, 2018). *Magnaporthe oryzae* responds to host nitro‐oxidative stress to suppress elevated ROS and host innate immunity, and RNS mitigation is connected to nitrogen metabolism (Marroquin‐Guzman *et al*., 2017). The necrotrophic fungus *Botrytis cinerea* exhibits complex cross‐talk of NO signalling with its plant hosts, and NO has important physiological consequences in the establishment of disease (Turrion‐Gomez and Benito, 2011). However, the roles of NO during the interaction between *V. dahliae* and its plant hosts have not been wellstudied.

In some pathogens, such as *Penicillium expansum* and *Colletotrichum coccodes*, NO can inhibit fungal growth by inducing the generation of ROS, which subsequently causes severe oxidative damage (Wang and Higgins, 2005; Lai *et al*., 2011). To overcome ROS stress, plant pathogens deploy both ROS scavenging enzymes and nonenzymatic antioxidants (Heller and Tudzynski, 2011; Segal and Wilson, 2018). Several fungal transcription factors are involved in the regulation of ROS‐responsive genes to resist an oxidative burst (Segal and Wilson, 2018). Among these transcription factors, Yap1, Atf1, and Skn7 are well studied in fungi and play important roles in oxidative responses (Moye‐Rowley, 2003; Fassler and West, 2011). Aside from their involvement of oxidative resistance, Yap1 and Atf1 are also responsive to RNS stresses. For instance, VdAtf1 is critical to an appropriate response to NO stress by regulating the transcription of genes involved in NO detoxification in *V. dahliae*, and an FgAtf1 mutant strain of *Fusarium graminearum* displayed increased sensitivity to NO (Tang *et al*., 2020). In *Schizosaccharomyces pombe*, Atf1 accumulates in the nucleus in response to nitrosative stress to promote stress tolerance (Kar *et al*., 2018), while in *Saccharomyces cerevisiae*, Yap1 also accumulates in the nucleus under nitrosative stress and mediates the changes in gene expression that correlate with RNS‐induced responses (Horan *et al*., 2006; Lushchak *et al*., 2010). Nevertheless, little is known about how Skn7 homologs mediate responses to RNS.

There are examples of overlapping or synergetic functions of Atf1, Yap1, and Skn7‐related homologs in fungi. FgAtf1 and FgSkn7 have overlapping functions in vegetative growth, sexual and asexual reproduction, stress responses, and pathogenesis in *F. graminearum* (Jiang *et al*., 2015). Both Atf1 and Pap1 (a fission yeast AP‐1‐like transcription factor) protect *S. pombe* against exogenous acute oxidative stress caused by glucose depletion (Madrid *et al*., 2004). In *Cochliobolus heterostrophus,* ChAP1 and Skn7 both function to regulate virulence and gene expression in response to oxidative stress (Shalaby *et al*., 2014). Nevertheless, it is not known whether these ROS‐responsive transcription factors have synergistic or overlapping effects with NO stress responsiveness.

VdAtf1 is essential to virulence and nitrogen metabolism but not H_2_O_2_ resistance (Fang *et al*., 2017; Tang *et al*., 2020) and VdSkn7 regulates microsclerotial development, H_2_O_2_ responsiveness, and virulence in *V. dahliae* (Tang *et al*., 2017). The aim of the present study was to systematically integrate the role of the three transcription factors VdSkn7, VdAtf1, and VdYap1 in nitrosative stress resistance, melanized microsclerotia formation, and virulence of *V. dahliae*. To investigate overlapping functions, a set of strains lacking single genes individually or double mutants of these genes were generated. Our results demonstrate that VdYap1 regulates ROS tolerance and that VdYap1 has overlapping functions with VdSkn7 in regulating nitrogen metabolism. VdSkn7 and VdAtf1 coregulate melanin formation and nitrogen metabolism. RNA‐Seq analyses of NO stress responses verified the overlapping and the distinct functions of the three transcription factors. Overall, the findings revealed convergent and distinctive functions of VdYap1, VdAtf1, and VdSkn7 in microsclerotial development, ROS/RNS responses, and virulence of *V. dahliae*, and shed light on the regulation of these functions in response to ROS/RNS in fungi.

## 2 RESULTS

### 2.1 The three *V. dahliae* transcription factors VdYap1, VdAtf1, and VdSkn7 differentially contribute to full virulence

The *V. dahliae* VdLs.17 genome (Klosterman *et al*., 2011) embraces *VDAG_08676*, *VDAG_02250,* and *VDAG_01588*, which are the orthologs of the transcription factors Atf1, Skn7, and Yap1 in yeast, respectively. Thus, these orthologs were named VdAtf1 (Fang *et al*., 2017), VdSkn7 (Tang *et al*., 2017), and VdYap1 in this study. To systematically reveal functions of these genes, single (∆*VdYap1*, ∆*VdAtf1*, and ∆*VdSkn7*) or double deletion mutants (∆*VdYap1*∆*VdAtf1*, ∆*VdYap1*∆*VdSkn7*, and ∆*VdAtf1*∆*VdSkn7*) were generated herein or previously (Table 1). Successful gene deletion transformants were confirmed by PCR and Southern blot (Figure S1). We chose one deletion strain from several putative mutants per deletion group for further experiments.

**TABLE 1:** Fungal strains used in this study

Strain name	Description	Reference
XS11	*Verticillium dahliae* wild‐type strain	Wang *et al*. (2013)
∆*VdAtf1*	Deleting *VdAtf1* (*VDAG_08676*) in XS11	Fang *et al*. (2017)
∆*VdSkn7*	Deleting *VdSkn7* (*VDAG_02250*) in XS11	Tang *et al*. (2017)
∆*VdYap1*	Deleting *VdYap1* (*VDAG_01588*) in XS11	This study
∆*VdYap1*∆*VdAtf1*	Deleting both *VdYap1* and *VdAtf1* in XS11	This study
∆*VdYap1*∆*VdSkn7*	Deleting both *VdYap1* and *VdSkn7* in XS11	This study
∆*VdAtf1*∆*VdSkn7*	Deleting both *VdSkn7* and *VdAtf1* in XS11	This study

**FIGURE 1:**
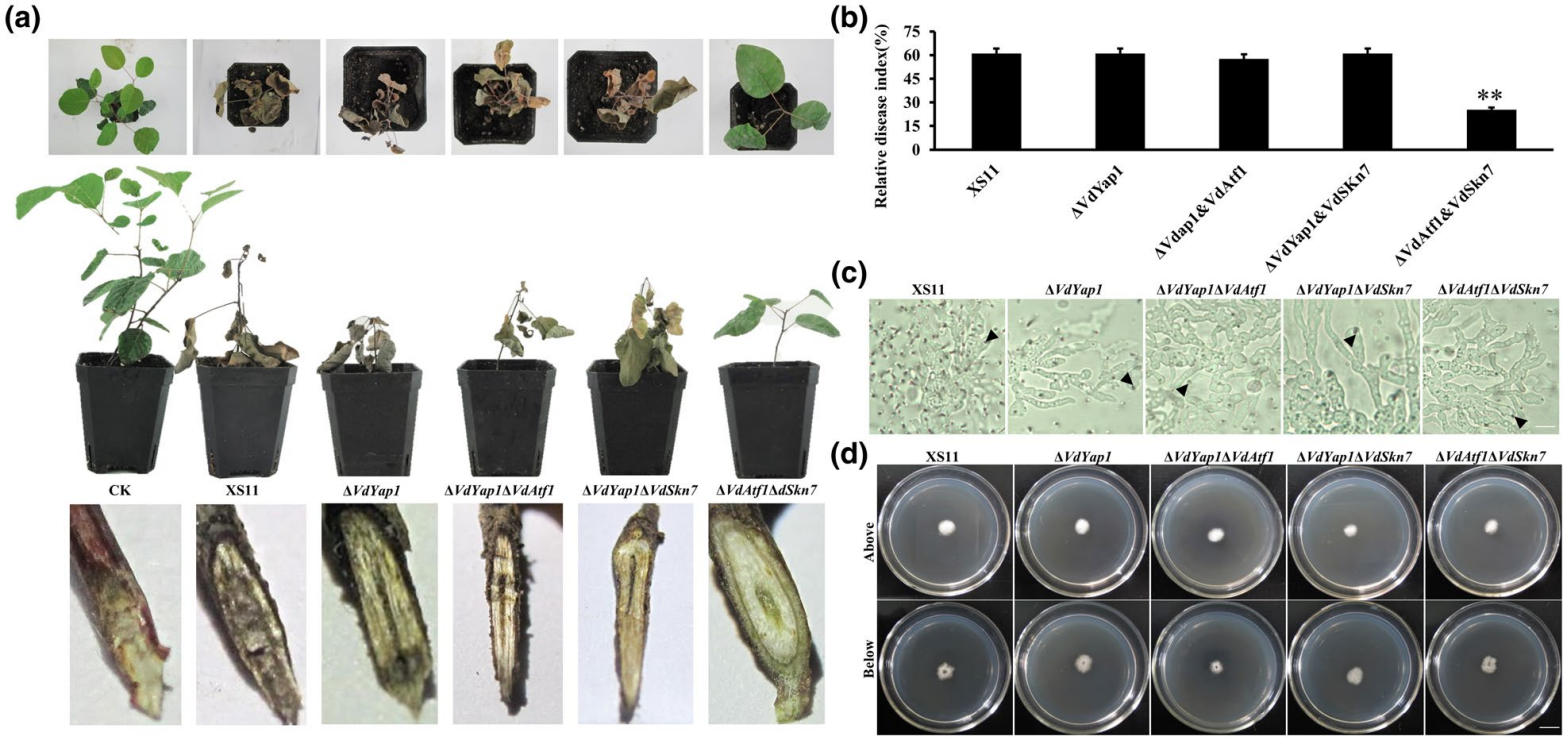
Pathogenicity tests of the Δ*VdYap1* strain of *Verticillium dahliae*. (a) Smoke tree (*Cotinus coggygria*) seedlings were inoculated with 10^6^ spores/ml conidial suspension of the wild type XS11, Δ*VdYap1*, Δ*VdYap1*Δ*VdAtf1*, Δ*VdYap1*Δ*VdSkn7*, and Δ*VdAtf1*Δ*VdSkn7* strains of *V. dahliae*. Controls (CK) were mock‐inoculated with water. All seedlings were placed in the greenhouse, and mature plant symptoms were photographed at 35 days postinoculation (dpi). Thirty seedlings were inoculated per strain. Photographs of the analyses of discoloration of shoots inoculated with each strain were taken at 35 dpi. (b) The relative disease index of seedlings inoculated with all strains. Error bars represent the standard deviations based on 10 independent replicates. Asterisks indicate significant differences (**p < .01, *p < .05). (c) Observation of penetration peg development after 3 days. The penetration pegs (from the hyphopodia) are indicated by the black arrows. (d) Strains of *V. dahliae* were grown on minimal medium overlaid with a cellophane layer for 3 days and photographed (above), and the plates were further incubated for 3 days after removal of the cellophane layer and photographed (below)

Pathogenicity assays on 1‐year‐old smoke tree seedlings were performed to evaluate their roles in virulence. Plants infected by the wild‐type strain XS11 exhibited typical verticillium wilt disease symptoms, with a relative disease index of 61% (Figure 1a,b). Interestingly, plants infected with Δ*VdYap1*, Δ*VdYap1*Δ*VdAtf1*, and Δ*VdYap1*Δ*VdSkn7* strains exhibited no distinguishable difference from those of the XS11 strain, whereas the double deletion of *VdSkn7* and *VdAtf1* clearly reduced the relative disease index by more than half (25%). Moreover, the Δ*VdAtf1*Δ*VdSkn7* strain was 11% lower than that of the Δ*VdSkn7* strain but similar to that of the Δ*VdAtf1* strain (Tang *et al*., 2017, 2020), indicating that the two transcription factors independently regulate pathogenicity. For further examination of the infection process, we used a cellophane membrane to observe infection structure formation. No differences were observed between any mutants and the XS11 strain in hyphopodia or penetration peg development (Figure 1c), and all strains penetrated the cellophane membrane at the same time (Figure 1d). These results suggest that VdYap1 is dispensable to virulence and VdSkn7 and VdAtf1 seem not to coregulate virulence in *V. dahliae*.

### 2.2 Deleting *VdYap1* attenuates microsclerotia formation

**FIGURE 2:**
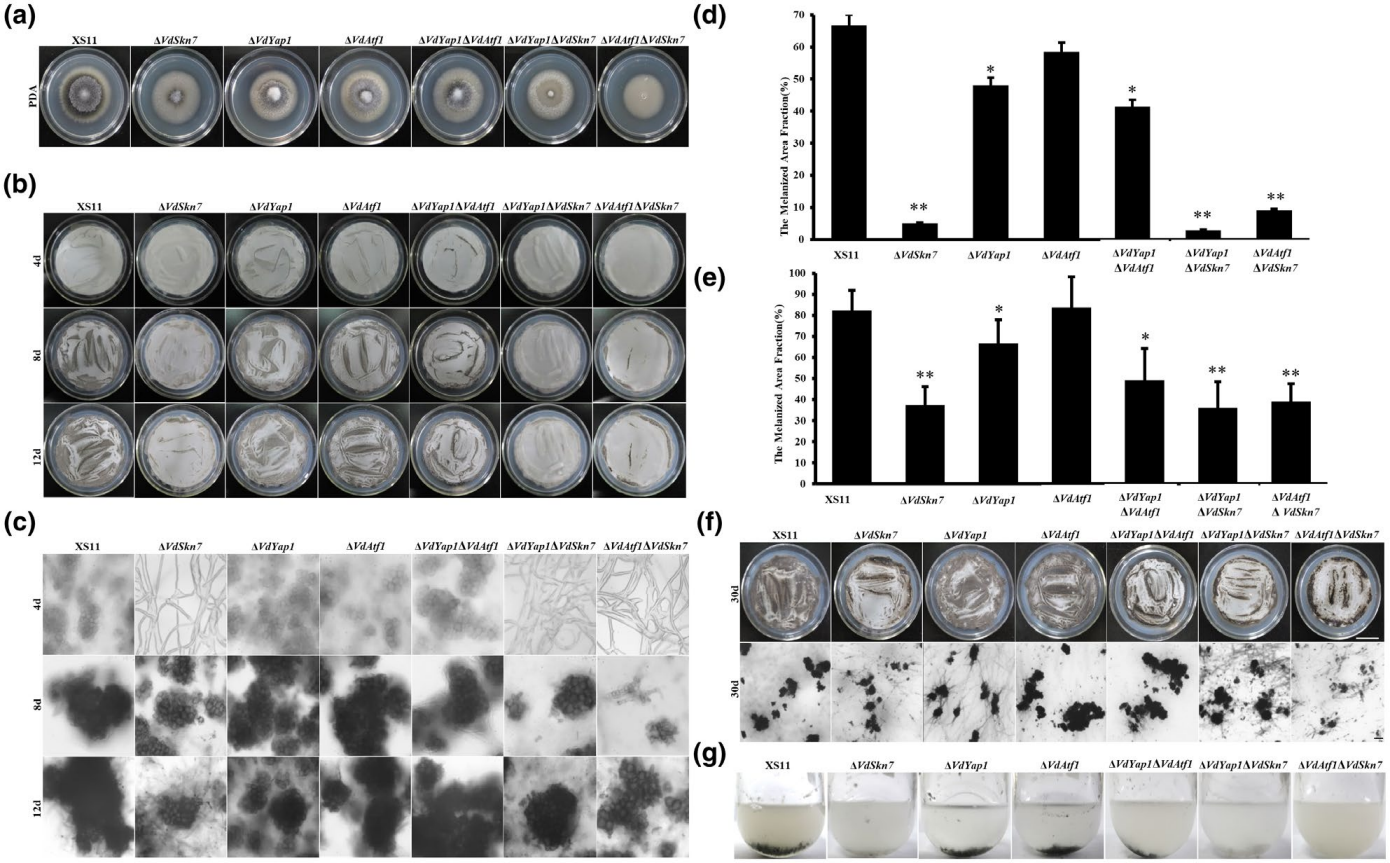
Microsclerotia formation of the Δ*VdSkn7*, Δ*VdYap1*, and Δ*VdAtf1* strains of *Verticillium dahliae*. (a) Ten‐day‐old cultures of the wildtype XS11, Δ*VdSkn7*, Δ*VdYap1*, Δ*VdAtf1*, Δ*VdYap1*Δ*VdAtf1*, Δ*VdYap1*Δ*VdSkn7*, and Δ*VdAtf1*Δ*VdSkn7* strains of *V. dahliae* grown on potato dextrose agar at 25°C. (b) Conidia (10^6^/ml) from each strain were spread over the cellulose membrane placed on basal medium (BM) plates and cultured at 25°C. Photographs were taken after 4, 8, and 12 days. (c) Microscopic observations of microsclerotia development in all strains after 4, 8, and 12 days. (d) and (e) Melanized fractions of the colonies were determined using ImageJ at 12 and 30 dpi. Error bars represent the standard deviations based on three independent replicates. Asterisks indicate significant differences (**p < .01, *p < .05). (f) Microsclerotia development on the cellulose membrane placed on BM plates. Photographs of plates and under microscopy were taken at 30 dpi. (g) Conidial suspensions (10^6^ spores) of the indicated strains of *V. dahliae* were shaken for 12 days at 25°C in liquid BM

At the later stage of the *V. dahliae* disease cycle, microsclerotia are typically produced in dying plant tissues (Fradin and Thomma, 2006; Klosterman *et al*., 2009). A previous study revealed that VdSkn7 is required for microsclerotia formation (Tang *et al*., 2017). In this study, all strains were cultured on potato dextrose agar (PDA) for 7 days, and the XS11 strain formed darkly melanized regions of microsclerotia production (Figure 2a). The Δ*VdYap1* and the Δ*VdYap1*Δ*VdAtf1* strain also exhibited the melanized regions, though these were smaller than those observed for the XS11 strain. In contrast, microsclerotia were completely absent in the Δ*VdYap1*Δ*VdSkn7* and Δ*VdAtf1*Δ*VdSkn7* strains. Examination of microsclerotia formation was undertaken in these strains using cellulose membranes overlaid on basal medium (BM) and a sample of the culture was removed for microscopic observations. Both the Δ*VdYap1* and Δ*VdAtf1* strains formed microsclerotia at 4 days postinoculation (dpi), similar to those formed in strain XS11, and formed mature black microsclerotia at 8 dpi, as did the Δ*VdYap1*Δ*VdAtf1* strain (Figure 2b,c). However, the Δ*VdYap1* and Δ*VdYap1*Δ*VdAtf1* strains exhibited reduced numbers of microsclerotia at 12 dpi (17% and 25% lower) and even at 30 dpi (18% and 33% lower) when compared with those of the Δ*VdAtf1* and XS11 strains, respectively (Figure 2d,e). Among the remaining mutant strains, the Δ*VdAtf1*Δ*VdSkn7* strain exhibited the slowest microsclerotial formation while the Δ*VdYap1*Δ*VdSkn7* strain was similar in this regard to the Δ*VdSkn7* strain. The number of microsclerotia in these strains was also reduced at 12 dpi, a decrease that remained significant at 30 dpi. After 30 days of culture, abundant mature melanized microsclerotia appeared in all strains except the Δ*VdAtf1*Δ*VdSkn7* strain, the analysis of which revealed a large number of immature microsclerotia, which were light grey or hyaline in appearance (Figure 2f). Analyses of all strains in shake cultures for 12 days in liquid BM revealed that the XS11, Δ*VdYap1*, and Δ*VdAtf1* strains formed melanized microsclerotia in similar colour and number while the Δ*VdYap1*Δ*VdAtf1* strain showed the same black colour but reduced numbers of microsclerotia (Figure 2g). The Δ*VdYap1*Δ*VdSkn7* strains formed microsclerotia at reduced numbers relative to the XS11 strain, and many of these were black like those of the wild type. The Δ*VdAtf1*Δ*VdSkn7* strain formed the fewest microsclerotia and those observed were not melanized. These results indicate that VdYap1 contributes to regulation of mature microsclerotia formation and VdAtf1 and VdSkn7 would coregulate melanin formation.

### 2.3 VdYap1 are involved in ROS resistance

**FIGURE 3:**
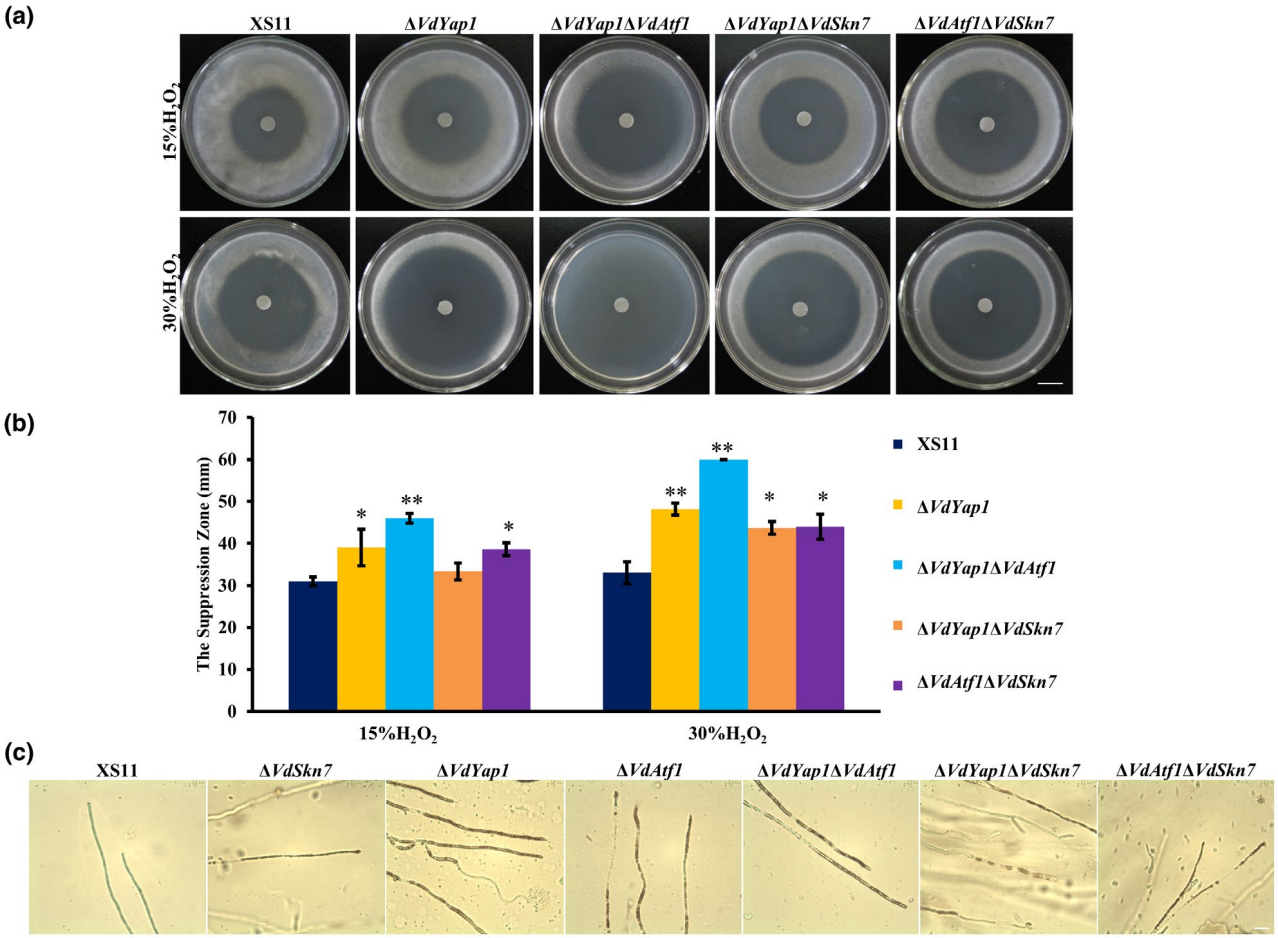
Oxidative stress responses of the Δ*VdYap1* strain and double deletion mutants of *Verticillium dahliae*. (a) Similar numbers of spores (10^7^ spores) of the wildtype XS11, Δ*VdYap1*, Δ*VdYap1*Δ*VdAtf1*, Δ*VdYap1*Δ*VdSkn7*, and Δ*VdAtf1*Δ*VdSkn7* strains of *V. dahliae* were added to potato dextrose agar (PDA) plates. Five millimetre diameter sterile filter paper disks were placed in the centre of the plates, to which 5 μl of H_2_O_2_ (15% and 30%) was added. The plates were incubated at 25°C for 2 days and the inhibition zones were recorded in millimetres. Scale bar = 0.85 cm. (b) The suppression zone of the above plates after 2 days' incubation. Error bars represent the standard deviations based on three independent replicates. Asterisks indicate significant differences (**p < .01, *p < .05). (c) Conidial suspensions of all strains (including Δ*VdSkn7* and Δ*VdAtf1* strains) were cultured on slides dipped on PDA at 25°C for 4 days. The hyphae of all strains were stained with 3,3′‐diaminobenzidine solution (pH 3.8) in the dark for 8 hr at room temperature and photographed under microscopy. Scale bar = 50 µm

To investigate the roles of VdYap1 with the VdAtf1 and VdSkn7 in ROS resistance, we examined the sensitivity of these strains to hydrogen peroxide (H_2_O_2_). We found that the Δ*VdYap1* mutant was much more sensitive to H_2_O_2_ than that of XS11 treated with 15% and 30% H_2_O_2_ (Figure 3a,b). Furthermore, when double deletion mutants were treated with 15% and 30% H_2_O_2_, we found that the inhibition rate of the Δ*VdYap1*Δ*VdAtf1* strain was 48% higher than that of the XS11 strain under 15% H_2_O_2_, and the Δ*VdYap1*Δ*VdAtf1* strain did not grow under 30% H_2_O_2_. The Δ*VdAtf1*Δ*VdSkn7* strain exhibited a 23% larger inhibition zone than that of the wild type XS11. However, the Δ*VdYap1*Δ*VdSkn7* strain showed hypersensitivity when treated with 30% H_2_O_2_. Meanwhile, all strains were cultured on PDA on a glass slide, and hyphae were stained by 3,3′‐diaminobenzidine (DAB) after culturing for 3 days for analysis of H_2_O_2_ degradation. All single and double mutants stained red, while the XS11 strain did not (Figure 3c). This result indicated that double deletion of *VdYap1* and *VdSkn7* directly affected H_2_O_2_ tolerance in *V. dahliae*, and unlike the XS11 strain, these mutants failed to neutralize H_2_O_2_, suggesting that VdYap1, VdAtf1, and VdSkn7 share a role in the mediation of redox balance in *V. dahliae*.

### 2.4 All three transcription factors are required for RNS resistance

**FIGURE 4:**
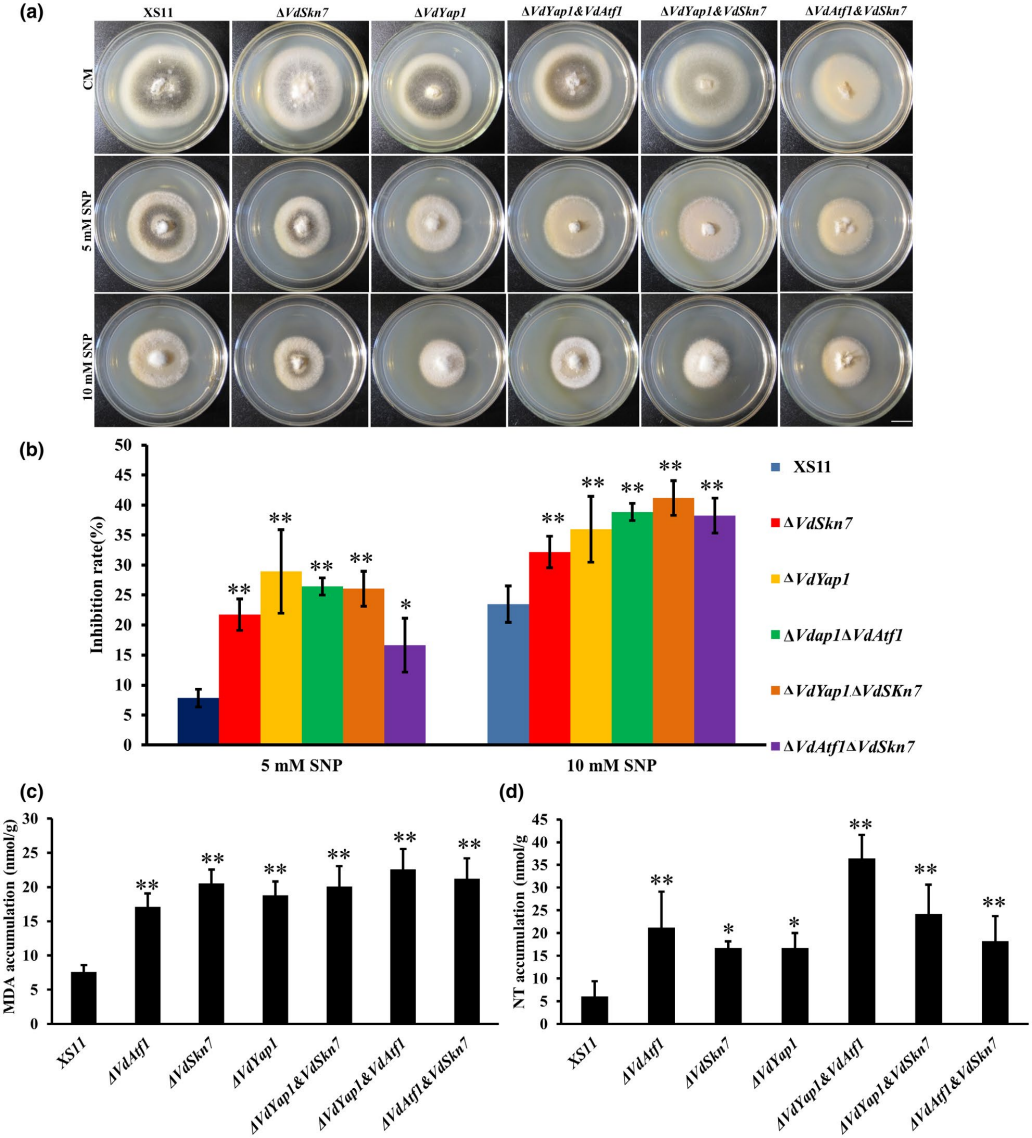
Functional analyses of the Δ*VdYap1* as well as double deletion mutants in nitric oxide stress response and in nitrogen utilization in *Verticillium dahliae*. (a) The wildtype XS11, Δ*VdSkn7*, Δ*VdYap1*, Δ*VdYap1*Δ*VdAtf1*, Δ*VdYap1*Δ*VdSkn7*, and Δ*VdAtf1*Δ*VdSkn7* strains of *V. dahliae* were cultivated on complete medium (CM) and CM with 5 and 10 mM sodium nitroprusside dehydrate (SNP) at 25°C. Photographs were taken after 10 days' incubation. (b) The suppression rate of all colonies derived from the strains of *V. dahliae* mentioned in (a) calculated after 10 days' incubation. All error bars represent the standard deviation based on at least three independent biological replicates. (c) and (d) Malondialdehyde (MDA) and nitrotyrosine (NT) accumulation of all strains treated 24 hr in CM with 10 mM SNP. Error bars represent the standard deviations based on three independent replicates. Asterisks indicate significant differences (**p < .01, *p < .05)

Our previous study demonstrated that VdAtf1 regulates nitrogen metabolism and NO resistance in *V. dahliae* (Tang *et al*., 2020). To examine the responsiveness of the other two transcription factors, we tested the sensitivity of all mutants to NO stress. In response to 5 and 10 mM sodium nitroprusside dehydrate (SNP) treatments, all of the mutant strains grew more slowly than the XS11 strain, and this was most evident in the Δ*VdYap1* strain as its inhibition zones were 21% and 12% higher than that of the XS11 strain when treated with 5 and 10 mM SNP, respectively (Figure 4a,b). Interestingly, knockout of *VdSkn7* also caused increased sensitivity to NO stress. The three double mutants showed significant hypersensitivity relative to the XS11 strain. The Δ*VdAtf1*Δ*VdSkn7* strain exhibited lower sensitivity to NO stress than the Δ*VdAtf1* and Δ*VdSkn7* single mutants when treated with 5 mM SNP, but all double mutants exhibited no differences as compared to the single deletion mutant strains at the 10 mM SNP concentration (Tang *et al*., 2020). We also measured malondialdehyde (MDA) and nitrotyrosine (NT) levels in all strains because both are makers for the presence of high levels of RNS (Marroquin‐Guzman *et al*., 2017). MDA and NT accumulated in the mycelia of all mutants compared to the XS11 strain (Figure 4c,d). This result illustrated that all three genes could regulate NO resistance and that they may have overlapping roles in NO resistance.

### 2.5 Comparative transcriptomic analysis of the XS11 strain exposed to NO stress

Strains Δ*VdAtf1*, Δ*VdSkn7*, and Δ*VdYap1* exhibited sensitivity to NO stress, but how each of these genes influences this sensitivity is uncertain. Therefore, we performed whole transcriptome analyses of each *V*. *dahliae* mutant strain and the XS11 strain with or without 10 mM SNP treatment. Forty‐two libraries, including all single and double mutants with or without NO stress treatment, as well as the strain XS11, were produced. After quality filtering, we obtained a total of 1,887.96 million reads from the 42 libraries, with an average of 44.95 million reads per sample and ranging from 38.08 to 54.38 million reads in each sample (Table S2). In total, 77.72% of the reads were mapped to the *V. dahliae* VdLs.17 reference genome (Klosterman *et al*., 2011) and 9,901 of the predicted genes were expressed. A two‐fold or greater change in expression and *p* < .05 was determined to be significant.

**FIGURE 5:**
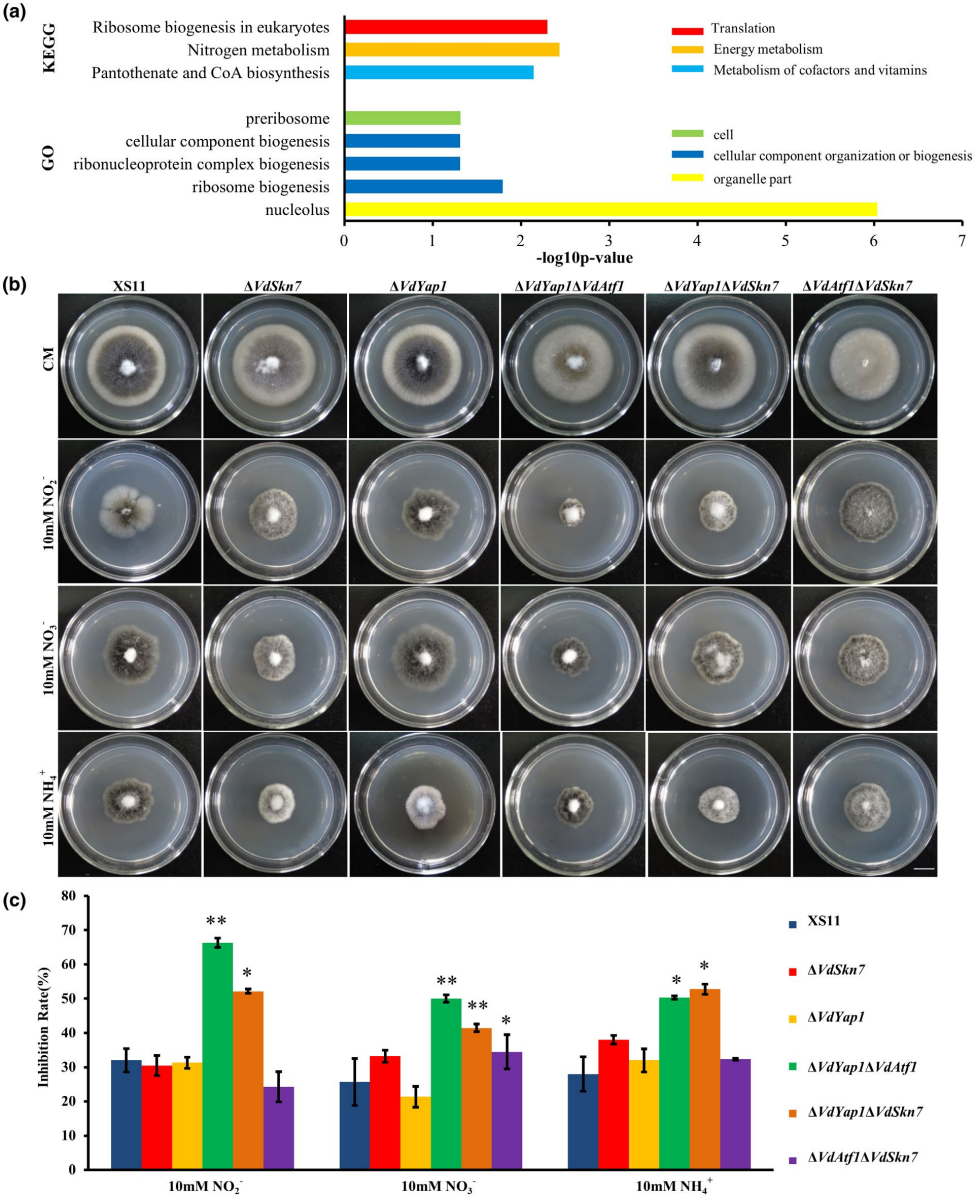
Analysis of differentially expressed genes (DEGs) in the wildtype XS11, Δ*VdAtf1*, Δ*VdYap1*, and Δ*VdSkn7* strains of *Verticillium dahliae* in response to nitric oxide stress. (a) Bar chart illustration of gene ontology and Kyoto Encyclopedia of Genes and Genomes pathway enrichments (*p* < .01) of all DEGs expressed in the XS11 strain with sodium nitroprusside dehydrate (SNP) treatment compared to those from XS11 strain without SNP treatment. (b) The wildtype XS11, Δ*VdSkn7*, Δ*VdYap1*, Δ*VdYap1*Δ*VdAtf1*, Δ*VdYap1*Δ*VdSkn7*, and Δ*VdAtf1*Δ*VdSkn7* strains of *V. dahliae* were grown for 10 days on complete medium and on defined minimal medium containing 1% (wt/vol) glucose (GMM) with 10 mM of the indicated sole nitrogen source (${\text{NO}}_{3}^{‐}$, ${\text{NO}}_{2}^{‐}$, or ${\text{NH}}_{4}^{+}$ at 25°C. Photographs were taken after 10 days' incubation of the media. (c) Inhibition rates of the above colonies after 10 days' incubation. Error bars represent the standard deviation based on three independent replicates. Asterisks indicate significant differences (**p < .01, *p < .05)

We identified the genes that were differentially expressed after NO stress in wild‐type XS11 strain compared to the strain grown in the absence of stress (control), and analyses of these data revealed 656 genes up‐regulated and 429 genes down‐regulated. Gene ontology (GO) enrichment revealed five terms while the Kyoto Encyclopedia of Genes and Genomes (KEGG) pathway enrichment revealed two significant pathways mainly related to metabolism, including pantothenate and CoA biosynthesis and nitrogen metabolism (Figure 5a).

The main inorganic nitrogen sources in nitrogen metabolism, such as nitrate (${\text{NO}}_{3}^{‐}$) and ammonium (${\text{NH}}_{4}^{+}$), can also serve as by‐products in NO metabolism, and NO is also a by‐product produced in nitrate metabolism (Chen *et al*., 2013; Marroquin‐Guzman *et al*., 2017). VdAtf1 is involved in nitrogen metabolism and NO response (Tang *et al*., 2020). Therefore, nitrogen utilization was tested in all strains. The growth on ${\text{NO}}_{2}^{‐}$, ${\text{NO}}_{3}^{‐}$, and ${\text{NH}}_{4}^{+}$ of the Δ*VdYap1* strain and the Δ*VdSkn7* strain was the same as that of the strain XS11 (Figure 5b,c) while the inhibition rate of the Δ*VdYap1*Δ*VdSkn7* strain was higher than that of the XS11 strain (34%, 24%, and 25%, respectively) as well as the single mutant strains Δ*VdYap1* (35%, 32%, and 22%, respectively) and Δ*VdSkn7* (36%, 17%, and 15%, respectively). The Δ*VdYap1*Δ*VdAtf1* strain also exhibited reduced nitrogen utilization, like the Δ*VdAtf1* strain. Interestingly, the Δ*VdAtf1*Δ*VdSkn7* strain restored the phenotype of the Δ*VdAtf1* strain, resulting in normal nitrogen metabolism. In summary, VdYap1 and VdSkn7 can coregulate nitrogen metabolism. However, deleting *VdSkn7* in the Δ*VdAtf1* strain had no influence on nitrogen utilization, suggesting that VdSkn7 has a negative impact on nitrogen utilization and this ability requires the participation of VdAtf1.

### 2.6 Differential gene expression individually regulated by VdAtf1, VdSkn7, and VdYap1 in response to NO stress

**FIGURE 6:**
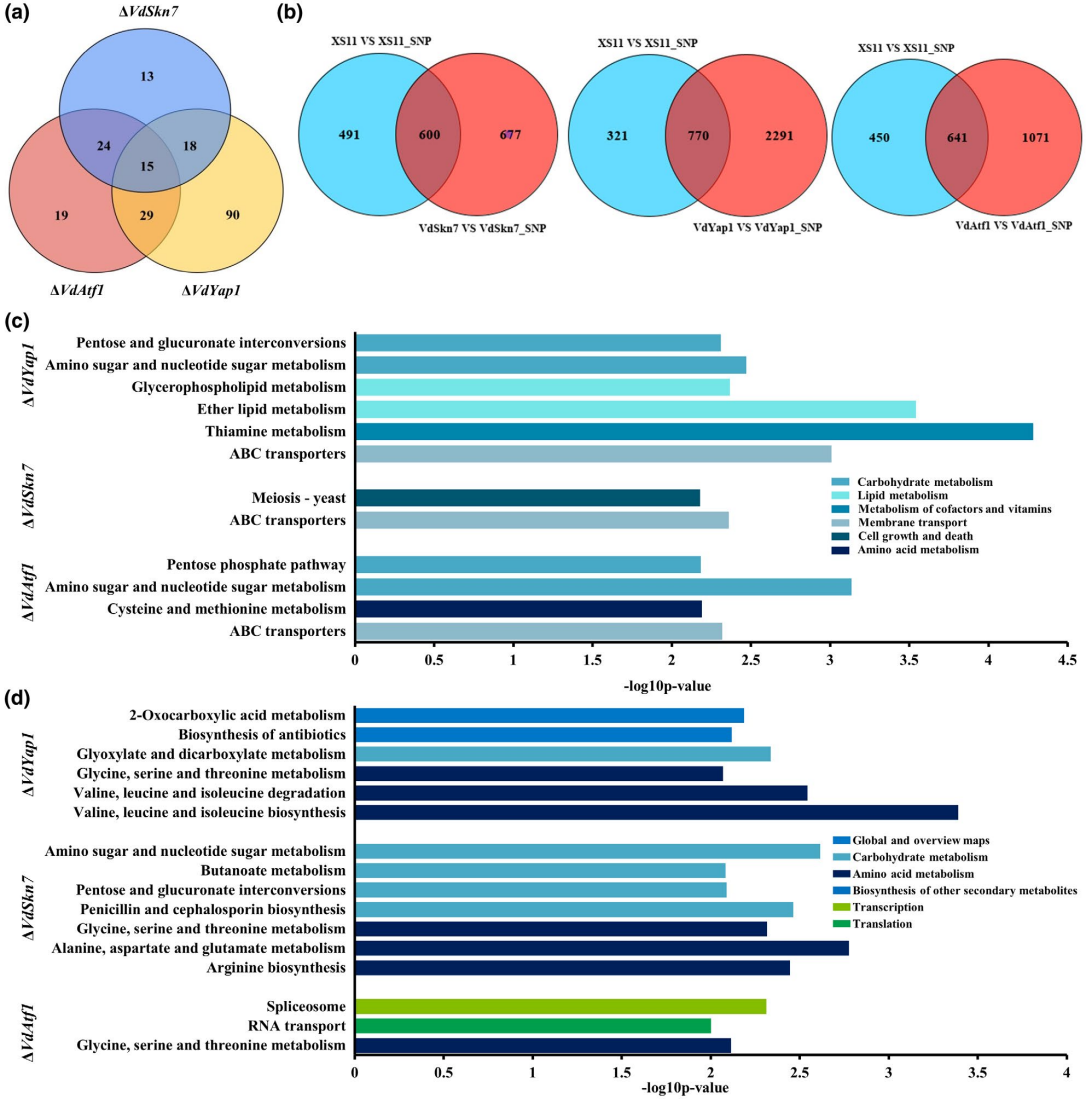
Genome‐wide transcriptome analysis of Δ*VdYap1*, Δ*VdAtf1*, and Δ*VdSkn7* strains of *Verticillium dahliae* in response to nitric oxide (NO) stress. (a) Venn diagram showing the number of genes controlled by each of the three single deletion mutants Δ*VdYap1*, Δ*VdAtf1*, and Δ*VdSkn7*. The sum of numbers in each circle represents the total number of genes between the two samples; overlapping parts of the circle represent commonly expressed genes in response to NO. (b) Venn diagram showing differentially expressed genes (DEGs) of three single mutants and the XS11 strain in NO stress. The sum of the numbers in each circle represents the total number of genes between the two samples; overlapping parts of the circle represent commonly expressed genes. (c) and (d) Bar chart illustration of Kyoto Encyclopedia of Genes and Genomes pathway enrichments (*p* < .01) of all DEGs depressed (c) or activated (d) by single gene deletion in response to NO stress

Because the three single mutants were sensitive to NO stress, we also wanted to identify genes dependent on their function under the nitrosative stress condition. Comparisons of gene expression in all strains with or without SNP treatment revealed 87 genes regulated by VdAtf1, 70 by VdSkn7, and 153 by VdYap1 under nitrosative stress (Figure 6a). The analysis showed that these genes were not expressed in the mutant strain with and without SNP treatment and in the XS11 strain grown in the absence of stress, but they were expressed in the XS11 strain with SNP treatment. Among these, 15 genes including *VDAG_02945* (chromate transporter) and *VDAG_05694* (copper chaperone for superoxide dismutase, CCS) were not expressed when any of the three transcription factors was knocked out, suggesting that the three transcription factors are equivalently important. In total, 19, 13, and 90 genes rely on the existence of VdAtf1, VdSkn7, and VdYap1, respectively, suggesting that VdYap1 has a major influence on NO stress response.

To further explore how the three transcription factors mediate NO stress responses in *V. dahliae*, we compared differentially expressed genes (DEGs) in the XS11 strain versus those of each single mutant strain, as shown in a Venn diagram (Figure 6b). GO enrichment analysis of down‐regulated genes revealed membrane compartment enriched categories such as “integral component of membrane” (GO: 0016021) and “intrinsic component of membrane” (GO:0031224) in all single mutants, suggesting that the main changes regulated by all three transcription factors mostly happened on the membrane (Table S3). The terms related to catalytic activity and metabolic process were also shared in all single mutants despite different GO terms enriched in different mutants. Moreover, transcription processes such as “transcription, DNA‐templated” (GO:0006351), “RNA biosynthetic process” (GO:0032774), and “nucleic acid‐templated transcription” (GO:0097659) were shared in the Δ*VdAtf1* and Δ*VdSkn7* strains, which seems to show both VdAtf1 and VdSkn7 restrain transcription activities to respond to NO stress. Both VdSkn7 and VdYap1 repressed genes involved in “protein kinase activity” (GO: 0004672). As for enrichments of significantly activated genes, “transmembrane transporter activity” (GO: 0022857) as well as “oxidoreductase activity” (GO: 0016491) were enriched in Δ*VdSkn7* strain, and 23 GO terms were significantly enriched in the Δ*VdYap1* strain, suggesting that VdYap1 up‐regulates more genes that participate in NO stress responses and these genes mostly concentrate in nuclear part and ribonucleoprotein complex (Table S4).

KEGG pathway enrichment was also analysed. DEGs related to ATP‐binding cassette (ABC) transporters and glycine, serine, and threonine metabolism were enriched in all single mutant strains, revealing that their common points are repressing transporter activity and stimulating amino acid metabolism (Figure 6c,d). All of the transcription factors control genes involved in metabolism in response to NO stress, though diverse metabolic pathways were enriched in different mutants. DEGs were enriched in the meiosis pathway only after deletion of *VdSkn7*, indicating that VdSkn7 might respond to NO resistance via depressing meiosis. All data showed that VdYap1, VdAtf1, and VdSkn7 not only have distinct regulation patterns but also share the same pathways in response to NO stress.

### 2.7 Transcriptomic profiles of the three double mutants Δ*VdYap1*Δ*VdAtf1*, Δ*VdYap1*Δ*VdSkn7*, and Δ*VdAtf1*Δ*VdSkn7* in response to NO treatment

To further probe the coregulation and overlapping functions of VdAtf1, VdSkn7, and VdYap1 in *V. dahliae*, gene expression in the double mutant strains Δ*VdYap1*Δ*VdAtf1*, Δ*VdYap1*Δ*VdSkn7*, and Δ*VdAtf1*Δ*VdSkn7* was also examined. For comparison, we concentrated on genes expressed only in the XS11 strain treated with SNP rather than in the XS11 strain without treatment and other mutants, revealing 12 genes in the Δ*VdYap1*Δ*VdAtf1* strain, 23 in the Δ*VdYap1*Δ*VdSkn7* strain, and 14 in the Δ*VdAtf1*Δ*VdSkn7* strain that were only expressed when each set of the two transcription factors was present, illustrating the coregulatory activity of the transcription factors.

**FIGURE 7:**
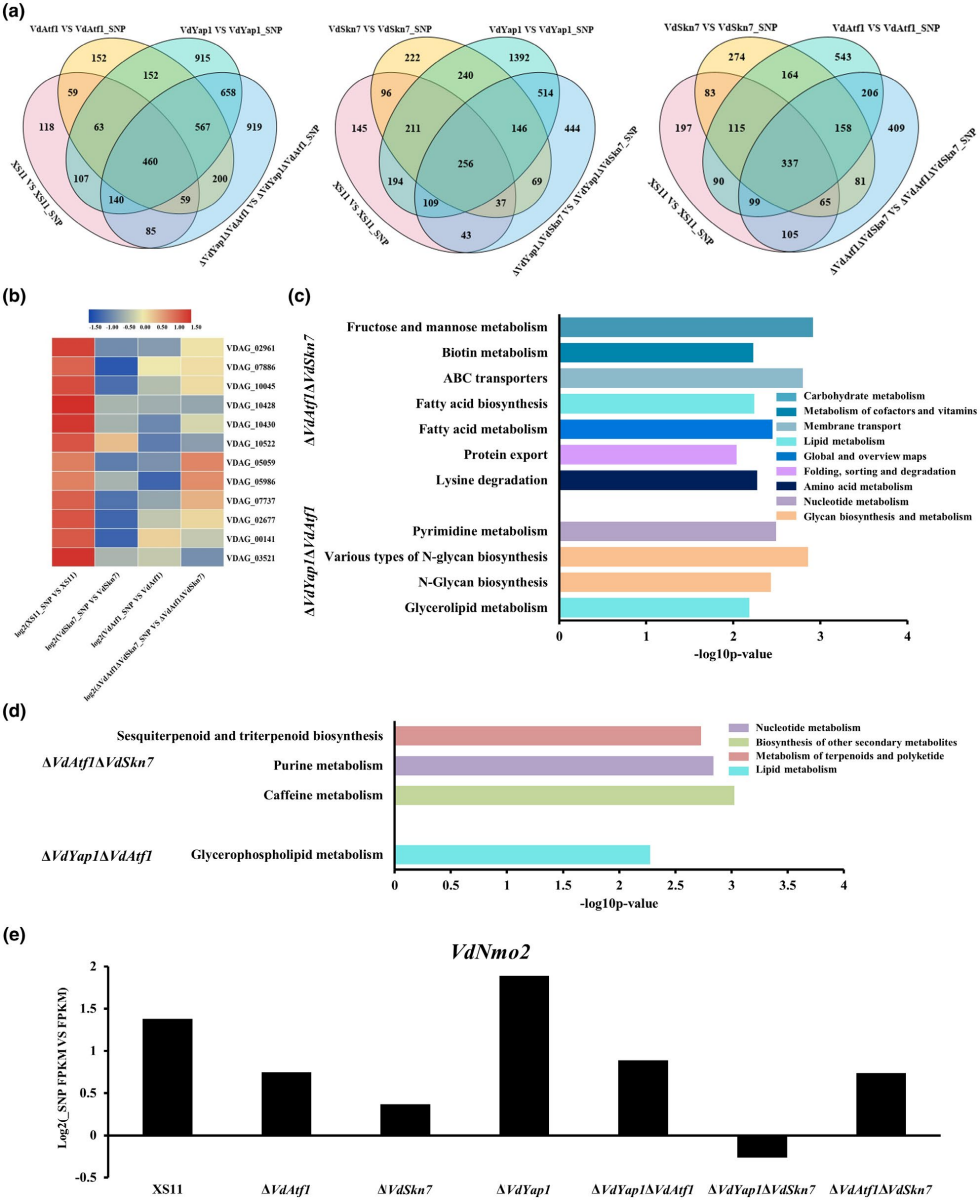
Genome‐wide transcriptome analysis of Δ*VdYap1*Δ*VdAtf1*, Δ*VdYap1*Δ*VdSkn7*, and Δ*VdAtf1*Δ*VdSkn7* strains of *Verticillium dahliae* in response to nitric oxide (NO) stress. (a) Venn diagram showing differentially expressed genes (DEGs) of the XS11 strain, double mutants Δ*VdYap1*Δ*VdAtf1*, Δ*VdYap1*Δ*VdSkn7*, and Δ*VdAtf1*Δ*VdSkn7*, and their related single mutants in NO stress. Circles with different colours represent each pairwise comparison between the wild‐type XS11 strain and the respective mutant strains Δ*VdSkn7*, Δ*VdYap1*, Δ*VdAtf1*, Δ*VdYap1*Δ*VdAtf1*, Δ*VdYap1*Δ*VdSkn7*, and Δ*VdAtf1*Δ*VdSkn7*. The sum of the numbers in each circle represents the total number of genes between the two samples; overlapping parts of the circle represent commonly expressed genes. (b) Heatmap of expression profiles of DEGs related to transcription in four mutants treated with 10 mM sodium nitroprusside dehydrate (SNP) as compared to those without SNP treatment. Log_2_(fragment per kilobase of exon model per million mapped reads[FPKM]) value was used and blue represents the negative value of log_2_(FPKM); red represents the positive value of log_2_(FPKM). Yellow represents the zero value of log_2_(FPKM). (c) and (d) Bar chart illustration of Kyoto Encyclopedia of Genes and Genomes pathway enrichments (*p* < .01) of all DEGs up‐regulated (c) or down‐regulated (d) in double gene deletion mutants in response to NO stress. (e) Gene expression difference of *VdNmo2* in all strains in response to NO stress

Comparisons of the DEGs in the XS11 strain versus every double mutant strain and the single mutants Δ*VdAtf1*, Δ*VdSkn7*, and Δ*VdYap1* is shown in the respective Venn diagrams (Figure 7a). Genes down‐regulated in the Δ*VdAtf1*Δ*VdSkn7* strain were significantly enriched in seven GO terms belonging to biological process, including transcription and some biosynthetic processes (Table S5). Meanwhile, genes related to “localization” (GO:0051179) that were down‐regulated in the Δ*VdYap1*Δ*VdSkn7* strain were also covered by the biological process. The Δ*VdYap1*Δ*VdAtf1* strain down‐regulated genes involved in molecular function such as “peptidase activity” (GO:0008233), “threonine‐type peptidase activity” (GO:0070003), and “threonine‐type endopeptidase activity” (GO:0004298). Among genes activated by knocking out double genes, GO enrichment revealed 11 GO terms in the Δ*VdAtf1*Δ*VdSkn7* strain, including the organelle part and cellular component organization or biogenesis, and revealed four GO terms in the Δ*VdYap1*Δ*VdSkn7* strain containing membrane part, which showed genes enriched in different locations in the two double mutants (Table S6). In the Δ*VdYap1*Δ*VdAtf1* strain, only the GO term “oxidoreductase activity” (GO:0016491) was enriched. The data illustrate that VdAtf1 and VdSkn7 have a complex influence on the NO response, while VdAtf1 and VdYap1 affect NO resistance by employing genes related to ROS resistance.

Thirty up‐regulated DEGs related to “oxidoreductase activity” were enriched in the Δ*VdYap1*Δ*VdAtf1* strain while they were not significantly expressed in either the Δ*VdYap1* strain or the Δ*VdAtf1* strain, suggesting that VdAtf1 and VdYap1 coregulate these genes. After comparisons of up‐regulated DEGs for oxidoreductase activity with the Δ*VdSkn7* strain, the gene encoding a glycerol‐3‐phosphate dehydrogenase (*VDAG_07740*)) was present in both lists, revealing that the three transcription factors have a complicated relationship in responding to NO stress. Ten DEGs related to “transcription, DNA‐templated” and “nucleic acid‐templated transcription” were enriched in the Δ*VdAtf1* strain and the same 10 DEGs plus *VDAG_00141* (RNA polymerase II transcription factor B subunit 5) were enriched in the Δ*VdSkn7* strain (Figure 7b). Among them, seven of the same DEGs were enriched in the Δ*VdAtf1*Δ*VdSkn7* strain. Six genes (*VDAG_02961*, *VDAG_07886*, *VDAG_10045*, *VDAG_10428*, *VDAG_10430*, and *VDAG_10522*) were highly expressed only when both *VdAtf1* and *VdSkn7* were present, and *VDAG_00141* was expressed only in the presence of *VdSkn7*. Moreover, *VDAG_03521* was remarkably expressed until both *VdAtf1* and *VdSkn7* were deleted. These data indicate that VdAtf1 and VdSkn7 play distinct and overlapping roles in NO response through genes related to nucleic acid biosynthesis.

Results of KEGG enrichment analyses were mixed for all three double mutant strains; none of the above pathways were enriched in the Δ*VdYap1*Δ*VdSkn7* strain. Pathways enriched in the other two double mutants were mainly concentrated in KEGG pathways belonging to various metabolisms, and metabolism pathways in repression were more than those in stimulation (Figure 7c,d). In addition, enrichment of repressed genes in the Δ*VdYap1*Δ*VdAtf1* strain included the terms of ABC transporters and protein export. All data demonstrated that VdYap1, VdAtf1, and VdSkn7 not only impact NO resistance, but also genetically interact with each other to regulate NO response.

In *M. oryzae*, Nmo2 (nitronate mono‐oxygenase) is required for mitigating nitro‐oxidative stress by repairing lipid damage either directly or indirectly from RNS (Marroquin‐Guzman *et al*., 2017). We were then curious about whether the expression profile of *VdNmo2* is linked to the responsiveness to NO stress in *V. dahliae*. The expression of *VdNmo2* was up‐regulated in all strains except the Δ*VdYap1*Δ*VdSkn7* strain under NO stress (Figure 7e). The up‐regulation of *VdNmo2* in the Δ*VdAtf1* and Δ*VdSkn7* strains was lower than that of the XS11 and Δ*VdYap1* strains, suggesting that deletion of *VdAtf1* and *VdSkn7* repressed the expression of *Nmo2* while knockout of *VdYap1* induced the expression under NO stress compared with the XS11 strain.

## 3 DISCUSSION

Fungi employ transcription factors to respond to many adverse environmental stresses, and the regulatory relationship among them is complicated. Herein, we found that VdYap1 is involved in ROS and RNS stress responses directly and contributes to microsclerotia development. Combined with our previous results (Tang *et al*., 2017, 2020), we demonstrated a general understanding of the overlapping and distinctive roles of three transcription factors, VdYap1, VdAtf1, and VdSkn7, in the regulation of nitrosative stress resistance, microsclerotia formation, and virulence in *V. dahliae*.

### 3.1 Functional cooperation of VdYap1, VdAtf1, and VdSkn7 in *V. dahliae*

Atf1, Skn7, and Yap1 are typical ROS‐responsive transcription factors whose mutual functional relationships in stress resistance have been extensively studied in fungi. In *V. dahliae*, we demonstrated that VdSkn7 is a key regulator of microsclerotia formation (Tang *et al*., 2017). In this study, these findings showed that VdYap1 and VdSkn7 both contribute to microsclerotia formation as well as H_2_O_2_ sensitivity, and VdAtf1 and VdSkn7 can both regulate virulence. These results suggest that the functions controlled by VdSkn7 are more diverse than the other two transcription factors, at least in relation to the few phenotypes examined. The phenotypes of double mutants were not more apparent than those of single mutants, revealing that these transcription factors have overlapping functions.

Apart from functional similarities, VdSkn7 interacts with VdYap1 to regulate nitrogen metabolism, and with VdAtf1 for melanin formation as well. VdAtf1 and VdYap1 also jointly affect the response to H_2_O_2_ stress in *V. dahliae*. In *F. graminearum*, glucose limitation in *S. pombe* induces oxidative stress, the detoxification of which depends on the cooperation of Pap1 and Atf1 (Madrid *et al*., 2004). Considering the oxidative stress response phenotypes of all strains in this study, it is difficult to explain the synergistic effect of VdYap1 and VdAtf1, though we may surmise two possible explanations. First, some VdYap1‐dependent genes essential for oxidative responses may also be activated by VdAtf1, in which case VdAtf1 is likely to partly offset oxidative sensitivity caused by deletion of *VdYap1*. In *S. pombe*, Atf1 can also regulate various Pap1‐dependent genes containing those encoding catalase and sulfiredoxin responsive to oxidative stress (Chen *et al*., 2008; Veal *et al*., 2014). Another possibility is that VdAtf1 and VdYap1 combine to form a heterodimer for transcription under oxidative stress. They belong to the bZIP transcription factor family and the bZIP domains contain a leucine zipper dimerization motif regulating dimerization (Jakoby *et al*., 2002). In addition, c‐Jun (Yap1 homolog) and ATF2 (Atf1 homolog) can form a heterodimer activating target gene expression to respond to many cellular and environmental signals in mammals (Liu *et al*., 2006).

### 3.2 Convergent and distinctive roles of VdYap1, VdAtf1, and VdSkn7 in RNS detoxification in *V. dahliae*

A high concentration of NO is toxic to cells by damaging proteins, lipids, and DNA directly or indirectly (Ischiropoulos and Gow, 2005). Fungi have developed detoxification strategies to counteract the damaging effects of NO and the ROS‐responsive transcription factors also play protective important roles. For example, in *S. pombe* Atf1 is a key player for the initiation of the S‐phase in the cell cycle, allowing a pause in replication to respond to nitrosative stress (Kar *et al*., 2018). In *S. cerevisiae* Yap1 is a key regulator in response to nitrosative stress by up‐regulating expression of superoxide dismutase and catalase (Lushchak *et al*., 2010). The current work demonstrates that VdAtf1, VdYap1, and VdSkn7 all play roles in NO stress responses and RNA‐Seq analyses indicated not only divergent but also shared regulation patterns.

The aberrant accumulation of MDA and NT in all mutants support the idea that the three transcription factors can denitrify nitrated alkanes and lipids to protect against nitro‐oxidative stress. In *M. oryzae*, Nmo2 played a crucial role in coping with nitro‐oxidative stress and the high concentration of MDA and NT may result from the misregulation of Nmo2 (Marroquin‐Guzman *et al*., 2017). In this study, we found that deletion of *VdAtf1* and *VdSkn7* down‐regulated expression of *VdNmo2* under NO stress, and when combined with our previous results we can infer both transcription factors are likely to mediate lipid damage repair to resist NO stress (Figure 7e). VdYap1 failed to influence the expression of *VdNmo2*, suggestive of unimpaired lipid damage repair. Furthermore, DEGs in the Δ*VdYap1* strain were enriched in metabolisms such as amino acid metabolism, lipid metabolism, and carbohydrate metabolism. We can infer that disturbances in metabolism may be responsible for NO sensitivity in the *VdYap1* deletion mutant. In *S. pombe*, pathway categories of meiosis and spliceosome were most affected under nitrosative stress (Biswas and Ghosh, 2015). Herein, genes related to meiosis were only down‐regulated in the Δ*VdSkn7* strain and those involved in RNA transport and spliceosome were only down‐regulated in the Δ*VdAtf1* strain, suggesting a novel relationship between the Skn7 homolog and meiosis, and suggesting that VdAtf1 can participate in transcription by potentially regulating the spliceosome. All single mutants also regulated genes corresponding to the ABC transporter category and metabolism to respond NO stress. ABC transporters, being present in virtually every living cell from prokaryotes and eukaryotes, hydrolyse ATP to transport a wide variety of compounds like toxic metabolites across extra‐ and intracellular membranes, playing important roles in regulating various physiological processes (Del Sorbo *et al*., 2000). In humans, the three transporters ABCB1, ABCC1, and ABCG2 (pleiotropic drug resistance [PDR] subfamily) have significant roles in multiple drug resistance, and among fungal ABC transporters the PDR subfamily is the only one involved in clinical antifungal resistance as reported so far (El‐Awady *et al*., 2017; Moreno *et al*., 2019). Thus, these types of genes encode products that can function in detoxification. When *V. dahliae* is exposed to NO stress, toxic metabolites would be induced because of disruptions to metabolism, requiring detoxification by transport out of the cell. Hence, VdAtf1, VdYap1, and VdSkn7 respond to NO stress by regulating ABC transporters.

Organisms have evolved subtle strategies to maintain redox balance within the cell for normal physiological activities (Segal and Wilson, 2018). Arbitrary changed expression of genes for oxidoreductase activity may cause cell damage owing to breaking the redox balance. Among genes related to oxidoreductase activity, *VDAG_08462* was most strikingly up‐regulated in the Δ*VdSkn7* strain, encoding ornithine acetyltransferase (ArgJ) which is involved in the synthesis of l‐arginine (Dou *et al*., 2011). Interestingly, stress‐induced arginine production can be used for NO production (Nishimura *et al*., 2010). Therefore, the deviant activation of VdArgJ induced by NO stress not only leads to a disorder of arginine biosynthesis but also results in excess NO production, causing damage. Another gene (*VDAG_07740*) related to oxidoreductase activity encodes glycerol 3‐phosphate dehydrogenase (GPD1), a key enzyme that plays an important regulatory role in the metabolism of glycerol (Albertyn *et al*., 1994). Single deletion of *VdSkn7* or double deletion of *VdAtf1* and *VdYap1* leads to its up‐regulation, causing abnormal glycerol accumulation. Over‐accumulation of intracellular glycerol may cause cell swelling, thereby leading to various morphological defects and ultimately growth inhibition (Randhawa *et al*., 2019).

Taken together, our results demonstrate convergent and distinctive functions of VdYap1, VdAtf1, and VdSkn7 in *V. dahliae*. Because the primary structures of each of these proteins are well conserved in fungi, the results also provide new insight on the potentially conserved roles of each of these proteins in response to ROS/RNS in fungi.

## 4 EXPERIMENTAL PROCEDURES

### 4.1 Fungal strain and culture conditions

The wild‐type *V. dahliae* strain XS11 was isolated from a smoke tree in Fragrant Hills, Beijing (Wang *et al*., 2013) and was used as a recipient to generate the different mutant strains used in this study. Conidial suspensions of all strains were stored long term at −80°C in 30% glycerol. Five‐day‐old vegetative hyphae collected from liquid compete medium (CM, 50 ml of 20 nitrate salts, 1 ml of 1,000 × trace element, 10 g glucose, 2 g peptone, 1 g yeast extract, 1 g casamino acids, and 1 ml of vitamin solution per litre) were used for genomic DNA extraction. Selection of strains resistant to the antibiotics hygromycin (Hyg; Sigma) and geneticin (G418; Sigma) was carried out by plating the respective strains on PDA plates (200 g potato, 20 g glucose, 15 g agar per litre) amended with 25 μg/ml hygromycin or 50 μg/ml geneticin, respectively. All strains were grown from a single‐spore culture on PDA at room temperature prior to experimentation, and mycelial masses used for inoculation were obtained using a punch (5 mm).

To test oxidative stresses, a 100 μl conidial suspension (10^7^ spores/ml) of each strain was spread onto PDA plates, and filter paper discs containing 5 μl of 15% and 30% hydrogen peroxide (H_2_O_2_) were placed onto the centre of each plate. The zone of growth inhibition was measured after 2 days. To observe ROS from hyphae, 0.2 μl conidial suspensions of all strains were cultured on slides dipped on PDA at 25°C for 4 days. Observations were made under light microscopy (DM2500; Leica) after staining with 1 mg/ml 3,3′‐diaminobenzidine (DAB; Sigma) solution (pH 3.8) in the dark for 8 hr at room temperature. For the nitrosative stress assay, all strains were grown for 10 days on CM amended with 5 or 10 mM NO donor sodium nitroprusside dehydrate (SNP; Sigma). As for growth tests on single nitrogen sources, all strains were cultured for 10 days on CM and on defined minimal medium containing 1% (wt/vol) glucose (GMM, 1.52 g KH_2_PO_4_, 0.52 g KCl, 0.152 g MgSO_4_.7H_2_O, 3 μM thiamine.HCl, 1.98 g glucose, 15 g agar per litre) with 10 mM of these sole nitrogen sources including nitrate (${\text{NO}}_{3}^{‐}$), nitrite (${\text{NO}}_{2}^{‐}$), and ammonium (${\text{NH}}_{4}^{+}$) at 25°C. The colony diameter was measured after 10 days. All the experiments were repeated three times.

To study microsclerotial production, conidia were harvested from cultures grown in liquid CM and 100 μl of a conidial suspension (10^6^ spores/ml) was spread on a cellulose membrane (Whatman, 80 mm diameter; pore size 0.22 μm) that had been overlaid on solid basal medium (BM, 10 g glucose, 0.2 g sodium nitrate, 0.52 g KCl, 0.52 g MgSO_4_.7H_2_O, 1.52 g KH_2_PO_4_, 3 μM thiamine.HCl, 0.1 μM biotin, 15 g agar per litre), then these plates were placed in the dark at 25°C. The contents and concentrations of the components of the solid BM were provided by Dr Katherine Dobinson, University of Western Ontario, Ontario, Canada. Distinct developmental stages were observed under light microscopy (DM2500, Leica) after 4, 8, 12, and 30 days, similar to those described previously by Xiong *et al*. (2014). For melanin formation, a 100 μl conidial suspension (10^6^ spores/ml) of each strain was added into liquid BM and shaken (150 rpm, 25°C) for 12 days. At 12 days, melanin formation was documented by photography. All the experiments were repeated three times.

Infection structures produced by *V. dahliae* were observed by analysing the fungus on cellophane membranes that were overlaid on the minimal medium (MM, 6 g NaNO_3_, 1.52 g KH_2_PO_4_, 0.52 g KCl, 0.52 g MgSO_4_, 20 mM l‐glutamic acid, 15 g agar per litre) plates. All strains were inoculated on cellophane at 25°C for 3 days, at which time the cellophane was removed from the surface of the plate and the plate was maintained for an additional 3 days to observe if colonies had formed after membrane removal. The colonies on the cellophane were rinsed to remove the residual conidia with sterile water, allowing observations of the remaining hyphae, hyphopodia, and penetration peg under light microscopy (DM2500; Leica). These experiments were repeated three times.

### 4.2 Construction, fungal transformations, and Southern blot

Targeted gene replacement of *VdYap1* was performed using the split‐marker method (Goswami, 2012) in *V. dahliae* strain XS11. To prepare the Yap1 knockout vector, the upstream and downstream flanking sequences of *VdYap1* were amplified by PCR using DNA of strain XS11 as the template with primer pair Yap1‐5Ffor and Yap1‐5Frev, and primer pair Yap1‐3Ffor and Yap1‐3Frev, respectively (Table S1). The geneticin resistance cassette was also amplified with primer pair Geneticinfor and Geneticinrev and then all sequences were connected to pMD19‐T vector plasmid (Takara) to obtain overlaps with the two flanking sequences. Both *VdYap1* flank amplicons were fused to the geneticin resistance cassette through fusion PCR. Subsequently, the overlapping DNA fragments, verified by sequencing (Thermo Fisher Scientific), were used for protoplast transformation directly. The *VdYap1* replacement construct was directly transformed into XS11 strain following established procedures described previously (Wang *et al*., 2013). To screen the *VdYap1* mutant, PCR amplifications were performed using primer pairs Yap1‐External‐F/R and Yap1‐Internal‐F/R.

To generate all double gene deletion mutants, the upstream and downstream flanking sequences of *VdYap1* and *VdAtf1* (Fang *et al*., 2017) were fused with the hygromycin‐resistant cassette using overlap PCR with primer pairs in Table S1. The resulting deletion constructs for *VdYap1* and *VdAtf1* were transformed into the protoplasts of the Δ*VdSkn7* strain. The *VdYap1* construct was also transformed into the Δ*VdAtf1* strain. Transformants were selected on TB_3_ medium (3 g yeast extract, 3 g casamino acids, 20% sucrose, and 0.7% agar per litre) supplemented with 25 μg/ml hygromycin. Successful double gene deletion transformants were screened by PCR with External‐F/Rs and Internal‐F/Rs of of each of the three genes.

Genomic DNA was extracted by a cetyltrimethylammonium bromide (CTAB) method. Southern blotting was performed to confirm the deletion of *VdYap1*, *VdAtf1*, and *VdSkn7* with the DIG High Prime DNA Labelling and Detection Starter Kit I (Roche) in accordance with the manufacturer's protocol. The probe fragment used for the Southern blot of VdYap1 single deletion was amplified from the *V. dahliae* strain XS11 genomic DNA with primers Yap1‐tzs and Yap1‐tzx and labelled with the DIG primer. The genomic DNA used for the Southern blot analysis was digested with *Eco*RV. The probe fragment used for the Southern blot of double deletion was amplified from the *HPH* gene with Hyg‐tzs and Hyg‐tzx and labelled with the DIG primer. The restriction enzyme *Eco*RI was used to digest the genomic DNA extracted from the wild‐type strain and double mutant strains.

### 4.3 Virulence assays

All strains were cultured in liquid CM medium for 5 days, then conidia were harvested by filtrating through two layers of Miracloth (Millipore) and diluted to 10^6^ conidia/ml by sterilized deionized water. Fifty 1‐year‐old smoke trees were root‐dipped in conidial suspensions of all strains for 10 min and were immediately replanted into sterilized soil in a greenhouse at 25°C. Control plants were mock‐inoculated with distilled water. All plants were observed after a period of 35 days and checked for morbidity with wilt symptoms. Shoots were cut after 35 days to observe vascular discoloration. Pathogenicity experiments were replicated three times.

### 4.4 Statistical analysis of melanized microsclerotia

The melanized area of the respective cultures was measured using ImageJ (Papadopulos *et al*., 2007) under the default settings (the threshold of all images was 178). Data were expressed as mean value ± standard error of the mean. Statistical analyses were performed using Student's *t* test. The *p* value < .05 was considered statistically significant in this study.

### 4.5 Assays of malondialdehyde and nitrotyrosine levels

The mycelia of all strains were obtained after culturing for 3 days in liquid CM, and for 3 days in CM prior to switching to different treatments such as CM with 10 mM SNP for 24 hr, and ground to a fine powder. All biochemistry measurements were determined using the spectrophotometric procedure described by the manufacturer (Solarbio).

### 4.6 RNA‐Seq and differential gene expression analysis

Fresh mycelia of all strains used for transcriptome profile analyses were grown in liquid CM at 25°C for 3 days before adding 10 mM SNP for 24 hr. The fungus was collected following one strain using a single‐layer of Miracloth (Millipore). All of the fungal samples were ground to a fine powder using a mortar and pestle in liquid nitrogen. RNA extraction was performed using TRIzol reagent (Invitrogen) and purified with the RNA Mini Kit (Ambion) in accordance with the manufacturer's instructions. After checking the quantity and quality of RNA using a NanoPhotometer spectrophotometer (Implen) and an RNA Nano 6000 Assay Kit of the Agilent Bioanalyzer 2100 system (Agilent Technologies), three biological replicates were sequenced by a BGISEQ‐500 platform (Beijing Genomics Institute). Raw reads were first processed using in‐house Perl scripts and clean reads were obtained by removing reads containing adapter and poly‐N sequences and low‐quality reads from the raw data. Data filtering was performed using SOAPnuke software (Beijing Genomics Institute). The reads were aligned to the *V. dahliae* reference genome (Klosterman *et al*., 2011) using Burrows‐Wheeler transform (Li and Dewey, 2011) to count the read numbers mapped to each gene and calculate gene expression.

The assembled transcripts in the final transcriptome were annotated by mapping them to several public databases. The DEGseq package (Wang *et al*., 2009) was used to provide statistical analyses for determining differential expression in digital gene expression data. Twelve libraries of deletion strains (∆*VdYap1*, ∆*VdAtf1,* ∆*VdSkn7*, ∆*VdYap1*∆*VdAtf1*, ∆*VdYap1*∆*VdSkn7*, and ∆*VdAtf1*∆*VdSkn7*) corresponding to the wild‐type strain XS11 with and without SNP treatment were used to detect DEGs. Significantly expressed genes with a |log_2_(fold‐change)| > 1 and *p* ≤ .05 were selected. The KEGG database (Hu *et al*., 2018) was used to identify enriched pathways by a two‐tailed Fisher's exact test to examine the enrichment of the differentially expressed individual gene products against all those identified. Significant enrichment was determined at *p* < .01. These pathways were classified into hierarchical categories according to the KEGG website (https://www.kegg.jp/kegg/kegg2.html).

## AUTHOR CONTRIBUTIONS

Y.W. and C.T. conceived the experiments. C.T. and X.J. performed the experiments. C.T., Y.W., and S.J.K. analysed the data. C.T., Y.W., and S.J.K. wrote the manuscript. All authors read and approved the manuscript.

## Data Availability

The data that support the findings of this study are available from the corresponding author upon reasonable request.
