# Supplementary material for: Convergent and distinctive functions of transcription factors VdYap1, VdAtf1, and VdSkn7 in the regulation of nitrosative stress resistance, microsclerotia formation, and virulence in Verticillium dahliae
Source: Mol Plant Pathol. 2020 Sep 20;21(11):1451–66. doi: 10.1111/mpp.12988 (PMC7549003; doi:10.1111/mpp.12988)
Supplement: Supplementary file 2 [file MPP-21-1451-s002.docx]

Table S1 Primers used in this study.

| **Primer name** | **Sequence** | **Use in this syudy** |
| --- | --- | --- |
| Atf1-5Ffor | CCAGGCATACCATTAGGAA | Atf1-5F flanking sequence |
| Atf1-5Frev | GATGGGTAAGGGTTCGTC |  |
| Atf1_3Ffor | CATAGGCACGGCAACACC | Atf1-3F flanking sequence |
| Atf1-3Frev | TCTCGTCCCAGACAGAGGC |  |
| Yap1-5Ffor | GCCTATGGGATACTGGCTAC | Yap1-5F flanking sequence |
| Yap1-5Frev | GAGGGAGAAAGACGGTGAT |  |
| Yap1_3Ffor | CTGCCCGCAAGCCTATGA | Yap1-3F flanking sequence |
| Yap1-3Frev | ACCAGGGACCGCCATTTT |  |
| Atf1-5Frev-O | GTCGTGACTGGGAAAACCCTGGCG GATGGGTAAGGGTTCGTC | Atf1-5F flanking overlapping sequence |
| Atf1_3Ffor-O | TCCTGTGTGAAATTGTTATCCGCT CATAGGCACGGCAACACC | Atf1-3F flanking overlapping sequence |
| Yap1-5Frev-O | GTCGTGACTGGGAAAACCCTGGCG GAGGGAGAAAGACGGTGAT | Yap1-5F flanking overlapping sequence |
| Yap1_3Ffor-O | TCCTGTGTGAAATTGTTATCCGCT CTGCCCGCAAGCCTATGA | Yap1-3F flanking overlapping sequence |
| Geneticinfor | GACGTTAACTGATATTGAAGGA | Geneticin cassette |
| Geneticinrev | GCTGGTGACGGAATTTTCAT |  |
| Hygfor | CGCCAGGGTTTTCCCAGTCACGAC | hygromycin cassette |
| Hygrev | AGCGGATAACAATTTCACACAGGA |  |
| En-F | AATATCACGGGTAGCCAACG | Geneticin overlapping sequence |
| Ge-R | TGAATGAACTGCAGGACGAG |  |
| Hy-R | GGATGCCTCCGCTCGAAGTA | hygromycin overlapping sequence |
| Yg-F | CGTTGCAAGACCTGCCTGAA |  |
| Yap1-tzs | CAACAACAGCAGCAACAG | Probe of VdYap1 Southern blot |
| Yap1-tzx | TTCGTCATCAGACCATCATT |  |
| Hyg-tzs | CACCGCGACGTCTGTCGAGAAG | Probe of double mutants Southern blot |
| Hyg-tzx | GGACGATTGCGTCGCATCGA |  |
| Yap1-Extrrnal-For | TGGATTTCTTCCCCTCCTCT | Yap1-External sequence |
| Yap1-Extrrnal-Rev | AGTGCATTGAGATCGGAACC |  |
| Yap1-Internal-For | CGCTCTCAACTCCAACAA | Yap1-Internal sequence |
| Yap1-Internal-Rev | GCTTCTTCGCAACCTTCT |  |
| Atf1-Extrrnal-For | GCGCTTGATCTGCCAGTC | Atf1-External sequence |
| Atf1-Extrrnal-Rev | CGCAAAGCCAAATACATACAG |  |
| Atf1-Internal-For | GCTTGCTAACCTCCAGACC | Atf1-External sequence |
| Atf1-Internal-Rev | CCTCCAGCCATCACTTGC |  |
