# Supplementary material for: Convergent and distinctive functions of transcription factors VdYap1, VdAtf1, and VdSkn7 in the regulation of nitrosative stress resistance, microsclerotia formation, and virulence in Verticillium dahliae
Source: Mol Plant Pathol. 2020 Sep 20;21(11):1451–66. doi: 10.1111/mpp.12988 (PMC7549003; doi:10.1111/mpp.12988)
Supplement: Supplementary file 3 [file MPP-21-1451-s003.docx]

Table S2 RNA sequencing statistics

| Sample | Total Clean Reads (M)^a^ | Total Mapping(%)^b^ | Uniquely Mapping(%)^c^ |
| --- | --- | --- | --- |
| VdAtf1&VdSkn7_1 | 43.42 | 91.53 | 72.77 |
| VdAtf1&VdSkn7_2 | 43.28 | 91.28 | 72.36 |
| VdAtf1&VdSkn7_3 | 44.59 | 91.22 | 72.49 |
| VdAtf1&VdSkn7_SNP_1 | 47.03 | 65.3 | 57.47 |
| VdAtf1&VdSkn7_SNP_2 | 45.96 | 64.37 | 56.3 |
| VdAtf1&VdSkn7_SNP_3 | 45.85 | 64.17 | 56.3 |
| VdAtf1_1 | 42.88 | 89.96 | 60.6 |
| VdAtf1_2 | 42.76 | 89.98 | 59.7 |
| VdAtf1_3 | 43.05 | 90.14 | 61.2 |
| VdAtf1_SNP_1 | 46.27 | 63.93 | 56.12 |
| VdAtf1_SNP_2 | 46.76 | 65.72 | 57.69 |
| VdAtf1_SNP_3 | 43.37 | 64.88 | 57.14 |
| VdSkn7_1 | 44.46 | 91.35 | 69.87 |
| VdSkn7_2 | 42.83 | 90.24 | 60.89 |
| VdSkn7_3 | 46.44 | 90.27 | 61.63 |
| VdSkn7_SNP_1 | 54.38 | 62.44 | 54.63 |
| VdSkn7_SNP_2 | 39.7 | 65.64 | 57.53 |
| VdSkn7_SNP_3 | 48.93 | 66.34 | 57.6 |
| VdYap1&VdAtf1_1 | 42.91 | 90.08 | 60.42 |
| VdYap1&VdAtf1_2 | 43.28 | 91.17 | 72.35 |
| VdYap1&VdAtf1_3 | 42.73 | 91.02 | 71.26 |
| VdYap1&VdAtf1_SNP_1 | 49.8 | 64.65 | 56.95 |
| VdYap1&VdAtf1_SNP_2 | 47.19 | 65.41 | 57.35 |
| VdYap1&VdAtf1_SNP_3 | 48.58 | 64.9 | 56.95 |
| VdYap1&VdSkn7_1 | 44.58 | 91.36 | 71.26 |
| VdYap1&VdSkn7_2 | 45.35 | 91.58 | 72.59 |
| VdYap1&VdSkn7_3 | 43.24 | 91.26 | 71.47 |
| VdYap1&VdSkn7_SNP_1 | 46.71 | 63.37 | 55.35 |
| VdYap1&VdSkn7_SNP_2 | 47.27 | 64.9 | 56.73 |
| VdYap1&VdSkn7_SNP_3 | 47.78 | 63.98 | 55.96 |
| VdYap1_1 | 42.82 | 89.94 | 59.87 |
| VdYap1_2 | 43.3 | 89.96 | 61.31 |
| VdYap1_3 | 44.42 | 89.95 | 61.15 |
| VdYap1_SNP_1 | 43.86 | 64.52 | 56.61 |
| VdYap1_SNP_2 | 38.08 | 66.83 | 58.54 |
| VdYap1_SNP_3 | 51.34 | 63.86 | 56.24 |
| XS11_1 | 43.38 | 91.28 | 70.96 |
| XS11_2 | 43.37 | 91.5 | 70.37 |
| XS11_3 | 42.98 | 90.86 | 69.25 |
| XS11_SNP_1 | 40.58 | 64.5 | 56.51 |
| XS11_SNP_2 | 48.32 | 64.59 | 56.74 |
| XS11_SNP_3 | 44.13 | 64.16 | 56.16 |

^a^ Raw data with low-quality reads, reads containing 39 adaptors, and reads containing two or more N bases removed.

^b^ Number of reads mapped to the reference genome within 2-bp mismatch.

^c^ Uniquely mapped = the number of reads mapped to the reference genome with unique sequence location.
