# Supplementary material for: Convergent and distinctive functions of transcription factors VdYap1, VdAtf1, and VdSkn7 in the regulation of nitrosative stress resistance, microsclerotia formation, and virulence in Verticillium dahliae
Source: Mol Plant Pathol. 2020 Sep 20;21(11):1451–66. doi: 10.1111/mpp.12988 (PMC7549003; doi:10.1111/mpp.12988)
Supplement: Supplementary file 4 [file MPP-21-1451-s004.docx]

Table S3 Enrichment terms of significantly activated genes (P-value < 0.01) in single mutants treated by NO stress.

| **Functional categories** | **GO Term** | **DEGs Number** | **P-value** |
| --- | --- | --- | --- |
| **Δ*VdAtf1*** | | | |
| **Biological_process** |  |  |  |
| GO:0006351 | transcription, DNA-templated | 10 | 0.003726098 |
| GO:0032774 | RNA biosynthetic process | 10 | 0.003726098 |
| GO:0097659 | nucleic acid-templated transcription | 10 | 0.003726098 |
| GO:0005975 | carbohydrate metabolic process | 40 | 0.000181785 |
| GO:0034654 | nucleobase-containing compound biosynthetic process | 34 | 0.007251236 |
| **Cellular_component** |  |  |  |
| GO:0016021 | integral component of membrane | 78 | 0.00546123 |
| GO:0031224 | intrinsic component of membrane | 78 | 0.005677956 |
| **Molecular_function** |  |  |  |
| GO:0070011 | peptidase activity, acting on L-amino acid peptides | 35 | 0.000101405 |
| GO:0008233 | peptidase activity | 35 | 0.000551469 |
| GO:0005215 | transporter activity | 21 | 0.005786155 |
| GO:0022857 | transmembrane transporter activity | 21 | 0.002254432 |
| GO:0008238 | exopeptidase activity | 13 | 0.000223857 |
| GO:0008236 | serine-type peptidase activity | 12 | 0.005955005 |
| GO:0017171 | serine hydrolase activity | 12 | 0.005955005 |
| GO:0015075 | ion transmembrane transporter activity | 11 | 0.004082225 |
| GO:0015318 | inorganic molecular entity transmembrane transporter activity | 10 | 0.008987045 |
| **Δ*VdSkn7*** | | | |
| **Biological_process** |  |  |  |
| GO:0006520 | cellular amino acid metabolic process | 13 | 0.008779456 |
| GO:0006351 | transcription, DNA-templated | 11 | 0.007348267 |
| GO:0032774 | RNA biosynthetic process | 11 | 0.007348267 |
| GO:0097659 | nucleic acid-templated transcription | 11 | 0.007348267 |
| **Cellular_component** |  |  |  |
| GO:0016020 | membrane | 93 | 0.000874427 |
| GO:0044425 | membrane part | 90 | 0.001225669 |
| GO:0016021 | integral component of membrane | 89 | 0.000410731 |
| GO:0031224 | intrinsic component of membrane | 89 | 0.00043226 |
| **Molecular_function** |  |  |  |
| GO:0004672 | protein kinase activity | 14 | 0.005280394 |
| **Δ*VdYap1*** | | | |
| **Biological_process** |  |  |  |
| GO:0005975 | carbohydrate metabolic process | 66 | 1.02E-08 |
| GO:1901137 | carbohydrate derivative biosynthetic process | 21 | 0.008577453 |
| GO:0016052 | carbohydrate catabolic process | 17 | 0.003951249 |
| GO:0005976 | polysaccharide metabolic process | 15 | 0.004177698 |
| **Cellular_component** |  |  |  |
| GO:0016020 | membrane | 70 | 0.000159404 |
| GO:0044425 | membrane part | 67 | 0.000503606 |
| GO:0016021 | integral component of membrane | 66 | 0.00025176 |
| GO:0031224 | intrinsic component of membrane | 66 | 0.000263084 |
| GO:0005576 | extracellular region | 22 | 0.000970641 |
| GO:0016020 | membrane | 290 | 7.70E-05 |
| GO:0044425 | membrane part | 282 | 6.04E-05 |
| GO:0031224 | intrinsic component of membrane | 279 | 4.03E-06 |
| GO:0016021 | integral component of membrane | 278 | 5.80E-06 |
| **Mmolecular_function** |  |  |  |
| GO:0016798 | hydrolase activity, acting on glycosyl bonds | 42 | 0.001341609 |
| GO:0004553 | hydrolase activity, hydrolyzing O-glycosyl compounds | 41 | 0.000985586 |
| GO:0004672 | protein kinase activity | 33 | 0.005914196 |
| GO:0003824 | catalytic activity | 403 | 0.000172089 |
