# Supplementary material for: Convergent and distinctive functions of transcription factors VdYap1, VdAtf1, and VdSkn7 in the regulation of nitrosative stress resistance, microsclerotia formation, and virulence in Verticillium dahliae
Source: Mol Plant Pathol. 2020 Sep 20;21(11):1451–66. doi: 10.1111/mpp.12988 (PMC7549003; doi:10.1111/mpp.12988)
Supplement: Supplementary file 5 [file MPP-21-1451-s005.docx]

Table S4 Enrichment terms of significantly depressed genes (P-value < 0.01) in single mutants treated by NO stress.

| **Functional categories** |  | | **GO Term** | **DEGs Number** | **P-value** |
| --- | --- | --- | --- | --- | --- |
|  | | ***VdAtf1*** | | | |
| **Cellular_component** |  | |  |  |  |
| GO:1990904 |  | | ribonucleoprotein complex | 20 | 0.006389625 |
| GO:0044429 |  | | mitochondrial part | 13 | 0.008719858 |
|  | | ***VdSkn7*** | | | |
| **Molecular_function** |  | |  |  |  |
| GO:0022857 |  | | transmembrane transporter activity | 19 | 0.009854677 |
| GO:0016491 |  | | oxidoreductase activity | 51 | 0.000430148 |
|  | | ***VdYap1*** | | | |
| **Biological_process** |  | |  |  |  |
| GO:0044237 |  | | cellular metabolic process | 303 | 0.001734054 |
| GO:0006807 |  | | nitrogen compound metabolic process | 267 | 0.000609348 |
| GO:0034641 |  | | cellular nitrogen compound metabolic process | 206 | 0.00247875 |
| GO:1901360 |  | | organic cyclic compound metabolic process | 184 | 0.006281075 |
| GO:0046483 |  | | heterocycle metabolic process | 180 | 0.001880163 |
| GO:0090304 |  | | nucleic acid metabolic process | 138 | 0.002410833 |
| GO:0016070 |  | | RNA metabolic process | 108 | 0.003792484 |
| GO:0006396 |  | | RNA processing | 52 | 0.001097942 |
| GO:0043412 |  | | macromolecule modification | 46 | 0.009494426 |
| GO:0022613 |  | | ribonucleoprotein complex biogenesis | 32 | 0.006551527 |
| GO:0034470 |  | | ncRNA processing | 28 | 0.003438554 |
| GO:0042254 |  | | ribosome biogenesis | 25 | 0.008447137 |
| GO:0008380 |  | | RNA splicing | 18 | 0.006144191 |
| GO:0000375 |  | | RNA splicing, via transesterification reactions | 17 | 0.002004034 |
| GO:0000377 |  | | RNA splicing, via transesterification reactions with bulged adenosine as nucleophile | 17 | 0.002004034 |
| GO:0000398 |  | | mRNA splicing, via spliceosome | 17 | 0.002004034 |
| GO:0009451 |  | | RNA modification | 14 | 0.009880988 |
| GO:0008643 |  | | carbohydrate transport | 12 | 0.001489426 |
| GO:0006575 |  | | cellular modified amino acid metabolic process | 11 | 0.003048603 |
| **Cellular_component** |  | |  |  |  |
| GO:0019012 |  | | virion | 10 | 0.005797656 |
| GO:0044423 |  | | virion part | 10 | 0.005797656 |
| GO:0044428 |  | | nuclear part | 60 | 0.006972276 |
| GO:1990904 |  | | ribonucleoprotein complex | 56 | 0.001106231 |
