# Supplementary material for: Convergent and distinctive functions of transcription factors VdYap1, VdAtf1, and VdSkn7 in the regulation of nitrosative stress resistance, microsclerotia formation, and virulence in Verticillium dahliae
Source: Mol Plant Pathol. 2020 Sep 20;21(11):1451–66. doi: 10.1111/mpp.12988 (PMC7549003; doi:10.1111/mpp.12988)
Supplement: Supplementary file 6 [file MPP-21-1451-s006.docx]

Table S5 Enrichment terms of significantly activated genes (P-value < 0.01) in double mutants treated by NO stress.

| **Functional categories** | **GO Term** | **DEGs Number** | **P-value** |
| --- | --- | --- | --- |
| **Δ*VdAtf1*Δ*VdSkn7*** | | | |
| **biological_process** |  |  |  |
| GO:0019438 | aromatic compound biosynthetic process | 12 | 0.000269131 |
| GO:0018130 | heterocycle biosynthetic process | 12 | 0.000398348 |
| GO:1901362 | organic cyclic compound biosynthetic process | 12 | 0.000673486 |
| GO:0034654 | nucleobase-containing compound biosynthetic process | 11 | 0.000363168 |
| GO:0006351 | transcription, DNA-templated | 8 | 0.0000816 |
| GO:0032774 | RNA biosynthetic process | 8 | 0.0000816 |
| GO:0097659 | nucleic acid-templated transcription | 8 | 0.0000816 |
| **Δ*VdYap1*Δ*VdAtf1*** | | | |
| **molecular_function** |  |  |  |
| GO:0008233 | peptidase activity | 44 | 0.000006140875 |
| GO:0004298 | threonine-type endopeptidase activity | 10 | 0.0000001461101 |
| GO:0070003 | threonine-type peptidase activity | 10 | 0.0000001461101 |
| **Δ*VdYap1*Δ*VdSkn7*** | | | |
| **biological_process** |  |  |  |
| GO:0051179 | localization | 19 | 0.007655661 |
