# Supplementary material for: Convergent and distinctive functions of transcription factors VdYap1, VdAtf1, and VdSkn7 in the regulation of nitrosative stress resistance, microsclerotia formation, and virulence in Verticillium dahliae
Source: Mol Plant Pathol. 2020 Sep 20;21(11):1451–66. doi: 10.1111/mpp.12988 (PMC7549003; doi:10.1111/mpp.12988)
Supplement: Supplementary file 7 [file MPP-21-1451-s007.docx]

Table S6 Enrichment terms of significantly depressed genes (P-value < 0.01) in double mutants treated by NO stress.

| **Functional categories** | **GO Term** | **DEGs Number** | **P-value** |
| --- | --- | --- | --- |
| **Δ*VdAtf1*Δ*VdSkn7*** | | | |
| **biological_process** |  |  |  |
| GO:0022613 | ribonucleoprotein complex biogenesis | 19 | 0.00000000407 |
| GO:0042254 | ribosome biogenesis | 18 | 0.000000000203 |
| GO:0006364 | rRNA processing | 10 | 0.00000774 |
| GO:0016072 | rRNA metabolic process | 10 | 0.00000996 |
| **cellular_component** |  |  |  |
| GO:0005730 | nucleolus | 13 | 0.0000000206 |
| GO:0030684 | preribosome | 10 | 0.0000000283 |
| GO:0044428 | nuclear part | 24 | 0.0000493 |
| GO:0031974 | membrane-enclosed lumen | 23 | 0.000013 |
| GO:0043233 | organelle lumen | 23 | 0.000013 |
| GO:0070013 | intracellular organelle lumen | 23 | 0.000013 |
| GO:0031981 | nuclear lumen | 22 | 0.000000802 |
| **Δ*VdYap1*Δ*VdAtf1*** | | | |
| **molecular_function** |  |  |  |
| GO:0016491 | oxidoreductase activity | 30 | 0.002592314 |
| **Δ*VdYap1*Δ*VdSkn7*** | | | |
| **cellular_component** |  |  |  |
| GO:0016021 | integral component of membrane | 76 | 0.00003670935 |
| GO:0031224 | intrinsic component of membrane | 76 | 0.00003869058 |
| GO:0016020 | membrane | 79 | 0.0000860133799999999 |
| GO:0044425 | membrane part | 77 | 0.00009362968 |
